# Supplementary material for: Identifying strengths and weaknesses of methods for computational network inference from single-cell RNA-seq data
Source: G3 (Bethesda). 2023 Jan 10;13(3):jkad004. doi: 10.1093/g3journal/jkad004 (PMC9997554; doi:10.1093/g3journal/jkad004)
Supplement: jkad004_Supplementary_Data [file jkad004_supplementary_data.zip › Supplemental_Figures_G3-2022-403885.pdf]

Supplementary figures

List of Figures

S1 Evaluation metrics for network inference on individual datasets. 3
S2 Effect of number of top edges selected on F-score based algorithm rankings 5
S3 Pearson correlation coefficients of method ranks between gold standards. 7
S4 Predictable TFs for the Tran (FBS) dataset. 9
S5 Predictable TFs for the Zhao dataset. 11
S6 Predictable TFs for the Shalek dataset. 13
S7 Predictable TFs for the Gasch dataset 15
S8 Jaccard similarity between top edge sets of inferred networks. 17
S9 F-score similarity between top edge sets. 19
S10 Comparison of Jaccard index and F-score as similarity metrics. 21
S11 Network inference utilizing gene expression alone compared to addition of priors and TFA . 23

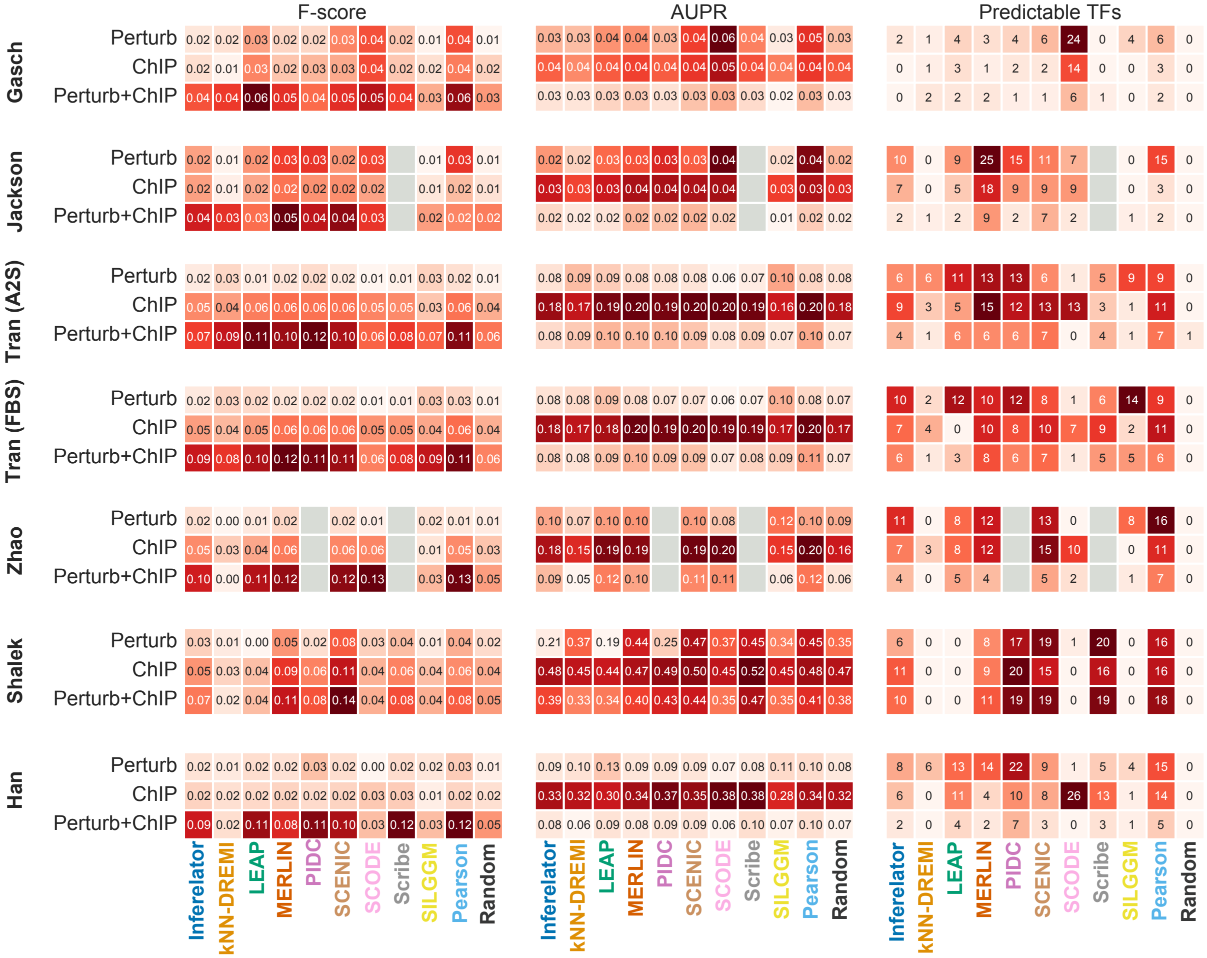
F-score
AUPR
Predictable TFs
Gasch
Jackson
Tran (A2S)
Tran (FBS)
Zhao
Shalek
Han
Perturb
ChIP
Perturb+ChIP
Inferelator
kNN-DREMI
LEAP
MERLIN
PIDC
SCENIC
SCODE
Scribe
SILGGM
Pearson
Random

Figure S1. Evaluation metrics for network inference on individual datasets. Heatmaps depict the values of each metric used for evaluating inferred networks. From left to right: F-score of top 5,000 edges, Area under the precision recall (AUPR) curve, and the number of predictable TFs. Algorithms are ordered alphabetically, followed by the Pearson and random networks.

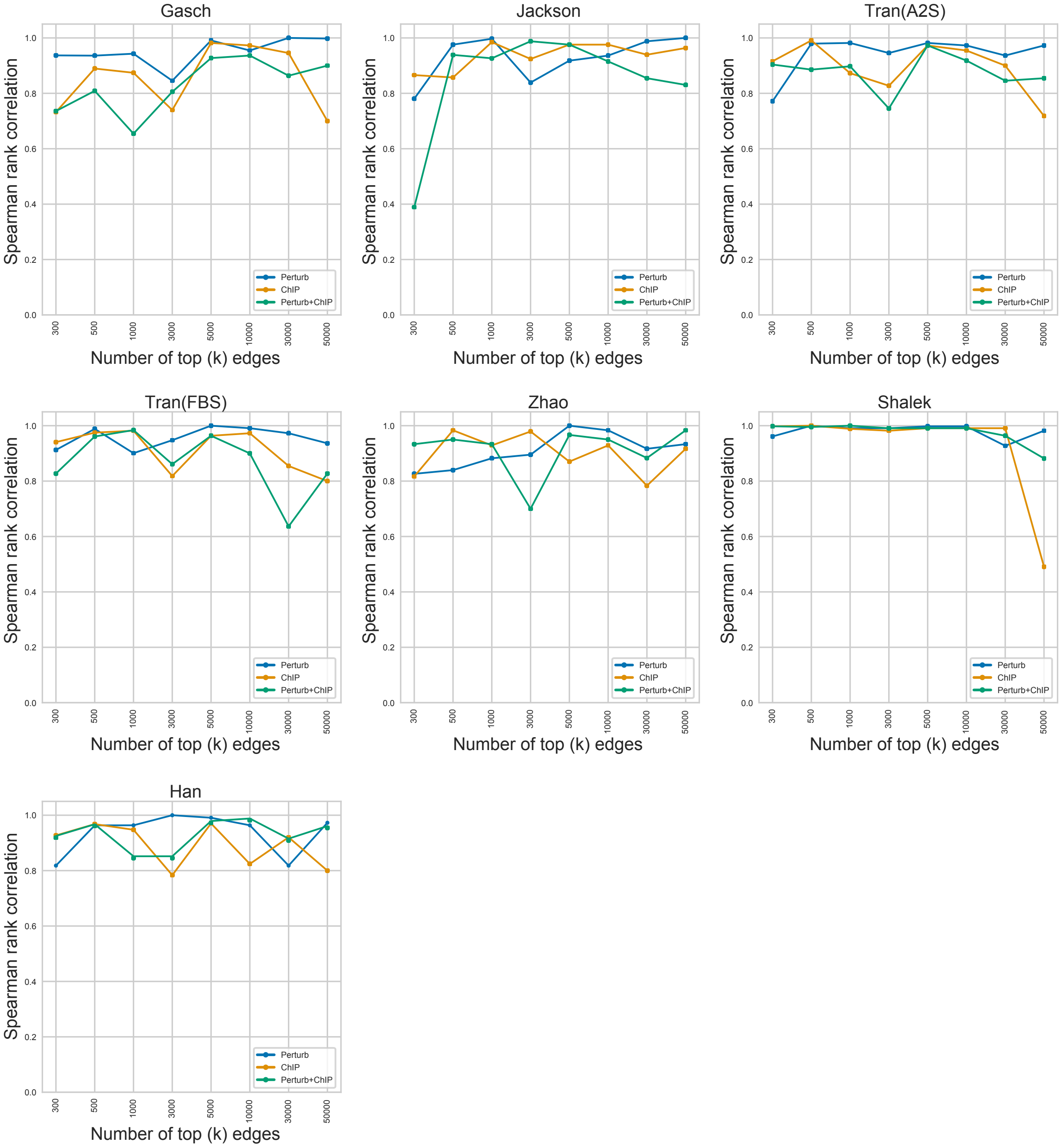
Gasch
Jackson
Tran(A2S)
Tran(FBS)
Zhao
Shalek
Han
Spearman rank correlation
Number of top (k) edges
Perturb
ChIP
Perturb+ChIP
0.0
0.2
0.4
0.6
0.8
1.0
300
500
1000
3000
5000
10000
30000
50000

Figure S2. Effect of number of top edges selected on F-score based algorithm rankings. We measured the F-score of each algorithm with respect to each experimentally derived network using the top 100, 300, 500, 1,000, 3,000, 5,000, 10,000, 30,000, and 50,000 edges. We computed the Spearman rank correlation between algorithm performances for each consecutive pair of edge sets, that is, the value corresponding to 300 on each graph shows the correlation between algorithm ranks when using the top 100 and top 300 edges. We found that across datasets, relative algorithm performance generally stabilized when using the top 5,000 edges and used this threshold for our analyses.

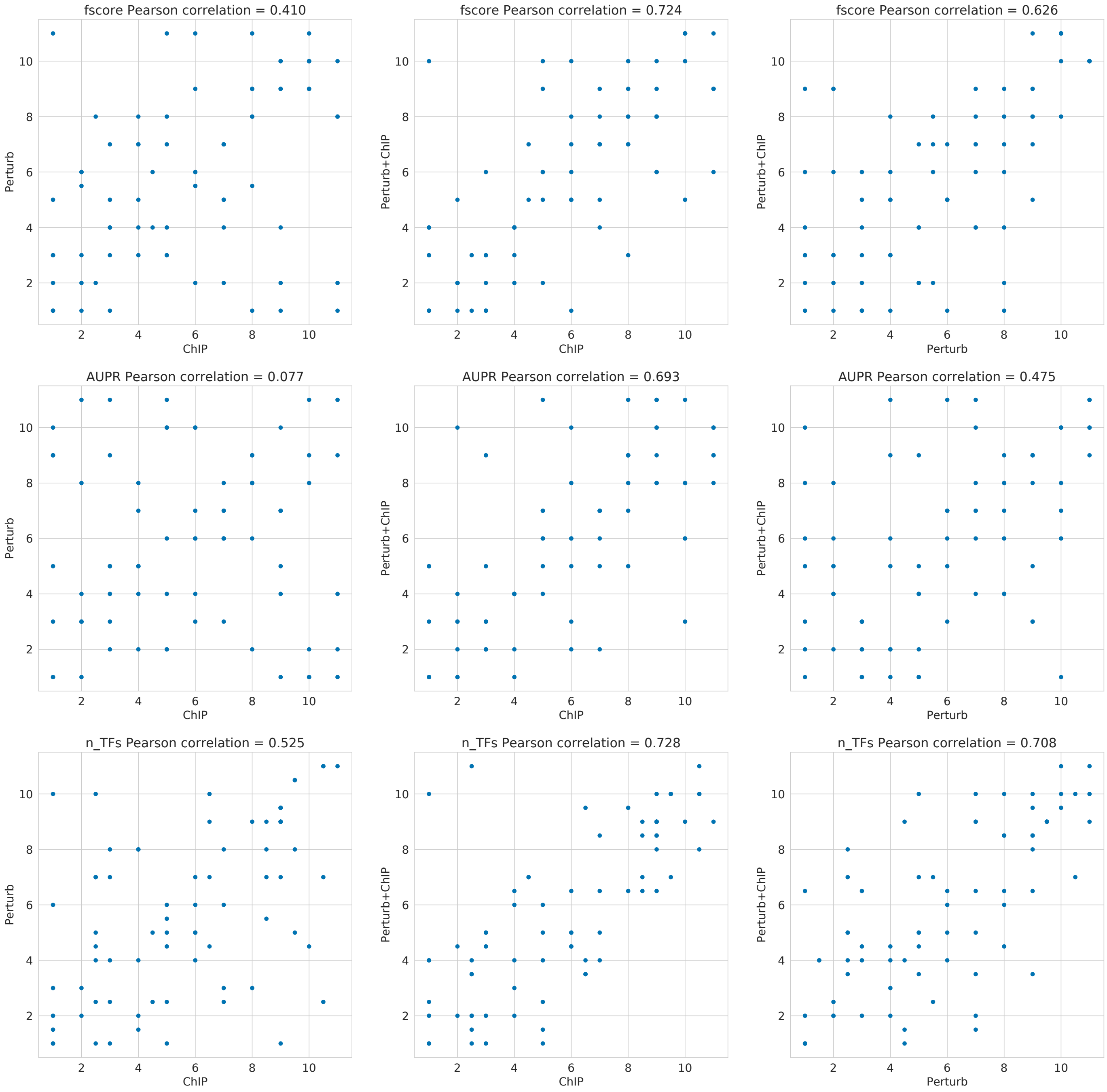
fscore Pearson correlation = 0.410
fscore Pearson correlation = 0.724
fscore Pearson correlation = 0.626
AUPR Pearson correlation = 0.077
AUPR Pearson correlation = 0.693
AUPR Pearson correlation = 0.475
n_TFs Pearson correlation = 0.525
n_TFs Pearson correlation = 0.728
n_TFs Pearson correlation = 0.708
ChIP
Perturb
Perturb+ChIP

Figure S3. Pearson correlation coefficients of ranks between gold standards when using different network inference evaluation metrics. Scatter plots show the correlations of algorithm ranks between pairs of gold standards (Perturb, ChIP, and Perturb+ChIP). The Pearson correlation coefficients are calculated based on ranks on the three evaluation metrics: AUPR, F-score, predictable TFs (n_TFs).

ChIP

	SCENIC	Pearson	MERLIN	PIDC	SCODE	LEAP	Scribe	Inferelator	kNN-DREMI	SILGGM
Number of predictable TFs	10	11	10	8	7	0	9	7	4	2
Sox2	1.6	1.6	1.6	1.6	1.3		1.4	1.5		
Esrrb	1.4	1.4	1.3	1.4				1.6		1.2
Nanog	1.5	1.4	1.3	1.4			1.2	1.4		
Sall4	1.7	1.9	1.8	2.2			1.8	1.9		
Nr0b1	2.8	2.7	2.1	2.4				3.1		1.5
Pou5f1	1.3	1.4	1.2	1.4			1.4			
Klf2	3.3	8.5		8.1			2.5	2.7	10.5	
E2f4		1.4	1.5		1.5			1.5		
Jarid2									2.6	
Mycn	1.4	1.3					1.3			
Zic3		3.1		3.1			5.1			
Myc	1.3		1.3		1.3					
Zic1										
Suz12									2.7	
Fosl2										
Gbx2										
Foxc1										
Zfx			1.8		1.5					
Chd1	2.8				2.2					
Dmrt1										
Id1										
Jun										
Sox15										
Stat3					1.6					
Phc1									2.4	
Tbx3			2.8							
Zfp42							1.6			
Klf4					1.5					
Otx2										
Trim28							1.2			
Klf5		11.8								
Lhx2										
Smad7										
Gata2										
Etv3										
Mybl2										

Perturb

	SCENIC	Pearson	MERLIN	PIDC	SCODE	LEAP	Scribe	Inferelator	kNN-DREMI	SILGGM
Number of predictable TFs	8	9	10	12	1	12	6	10	2	14
Sox2	1.5	1.4	1.5	1.6		1.7	1.3	1.7		1.7
Esrrb	3.0	2.2	2.9	2.4		1.4	3.4	3.5		2.6
Nanog	2.1	2.1	2.0	2.1		2.1	1.7	2.4		2.3
Sall4		1.6	1.5	1.9		1.7	1.5	1.7	2.0	1.8
Nr0b1	4.7		3.9			1.7		5.3		3.6
Pou5f1		1.7	1.7	1.6		1.4				1.3
Klf2										
E2f4										
Jarid2	2.4	3.0		2.7			1.9	2.7		
Mycn										
Zic3										
Myc										
Zic1	4.3		4.0	12.2				6.8		2.6
Suz12					3.5		4.6			
Fosl2		2.5		2.3		1.9		1.7		
Gbx2		1.9		2.0		2.3				1.6
Foxc1	2.2		2.0	3.4				2.4		
Zfx										
Chd1										
Dmrt1		1.7	1.5							1.2
Id1				8.6		22.9		4.8		
Jun	2.3			2.8		2.1				
Sox15						2.2				2.3
Stat3										
Phc1										
Tbx3										
Zfp42										
Klf4										
Otx2			2.6							2.0
Trim28										
Klf5										
Lhx2										2.2
Smad7						1.6				
Gata2									1.4	
Etv3										10.2
Mybl2										2.6

Number of predictable TFs

0.0	4.0	8.0	12.0	16.0	20.0

Fold enrichment

0.0	1.0	2.0	3.0	4.0	5.0

Figure S4. Predictable TFs for the Tran (FBS) dataset. Heatmaps show the enrichment of each transcription factor's target set from a perturbation-based (left) or ChIP-based (right) experimentally derived gold standard network in an inferred network. Columns are ordered based on overall ranking of methods. Individual white cells indicate TFs that were not considered as a predictable TF by a method. An entire row of white cells indicates the TF did not appear in one of the two experimentally derived networks.

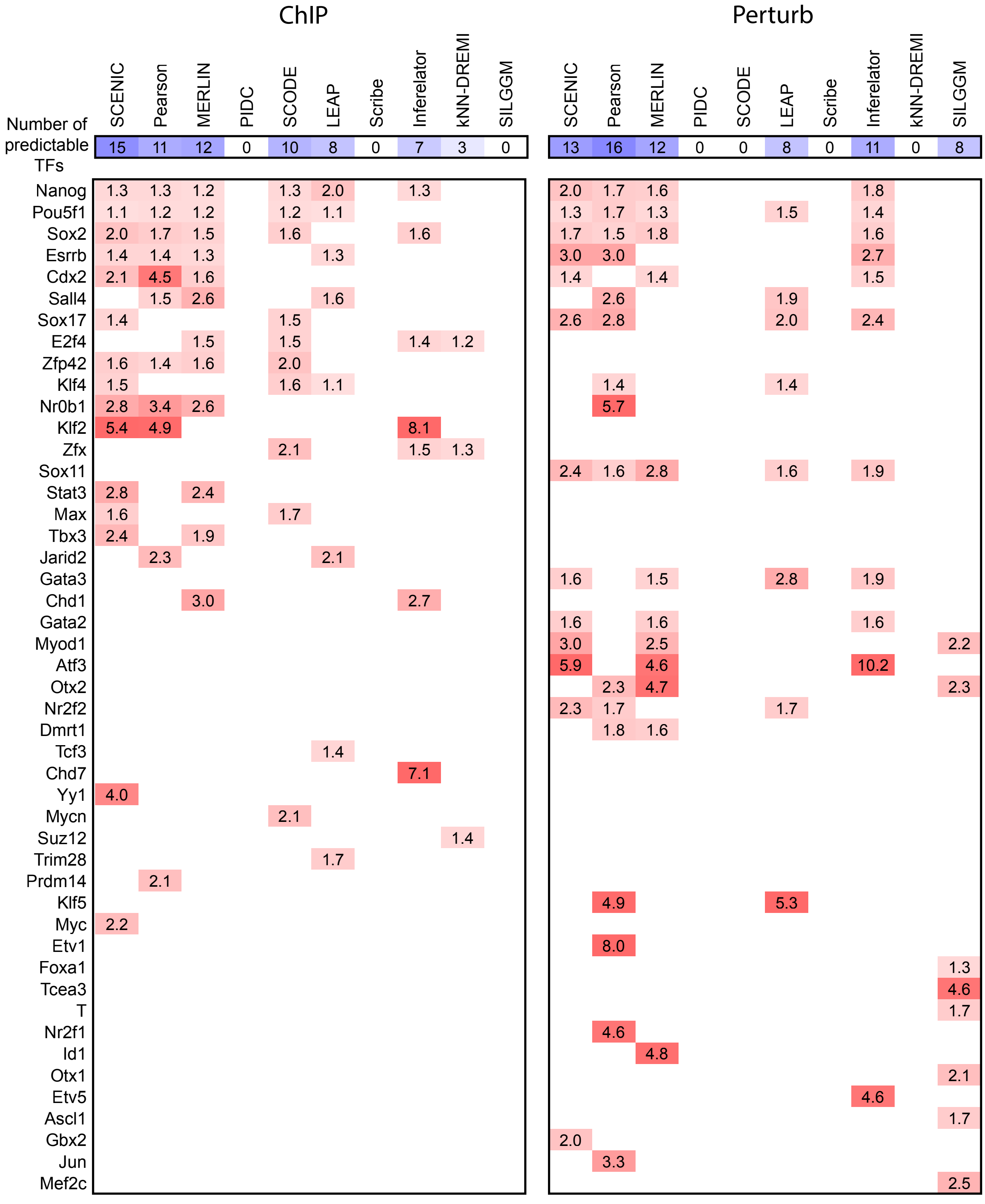
ChIP
Perturb
SCENIC
Pearson
MERLIN
PIDC
SCODE
LEAP
Scribe
Inferelator
kNN-DREMI
SILGGM
Number of predictable TFs
15 11 12 0 10 8 0 7 3 0
13 16 12 0 0 8 0 11 0 8
Nanog
Pou5f1
Sox2
Esrrb
Cdx2
Sall4
Sox17
E2f4
Zfp42
Klf4
Nr0b1
Klf2
Zfx
Sox11
Stat3
Max
Tbx3
Jarid2
Gata3
Chd1
Gata2
Myod1
Atf3
Otx2
Nr2f2
Dmrt1
Tcf3
Chd7
Yy1
Mycn
Suz12
Trim28
Prdm14
Klf5
Myc
Etv1
Foxa1
Tcea3
T
Nr2f1
Id1
Otx1
Etv5
Ascl1
Gbx2
Jun
Mef2c

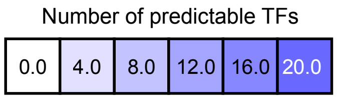
Number of predictable TFs
0.0
4.0
8.0
12.0
16.0
20.0

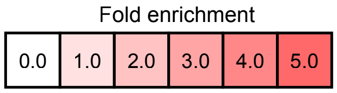
Fold enrichment
0.0
1.0
2.0
3.0
4.0
5.0

Figure S5. Predictable TFs for the Zhao dataset. Heatmaps show the enrichment of each transcription factor's target set from a perturbation-based (left) or ChIP-based (right) experimentally derived network in an inferred network. Columns are ordered based on overall ranking of methods. Individual white cells indicate transcription factors that were not considered as a predictable TF by a method. An entire row of white cells indicates the TF did not appear in one of the two experimentally derived networks.

ChIP

	SCENIC	Pearson	MERLIN	PIDC	SCODE	LEAP	Scribe	Inferelator	kNN-DREMI	SILGGM
Number of predictable TFs	15	16	9	20	0	0	16	11	0	0
Atf3	1.3	1.3	1.2	1.4			1.3	1.2		
Rel	1.5	1.5	1.3	1.8			1.5	1.5		
Stat1	1.1	1.1	1.2	1.2			1.1	1.1		
Nfkb1	2.0	2.0	1.8	2.3			1.5	2.0		
Stat3	1.1	1.2	1.2	1.3			1.4	1.2		
Relb	1.5	1.5	1.4	1.7			1.6	1.4		
Stat2	1.1	1.1		1.2			1.2	1.1		
Egr2	1.2	1.2		1.2			1.2	1.2		
Cebpb	1.1	1.1		1.0			1.1			
Irf1	1.1	1.1		1.2			1.1			
Junb	1.1	1.1	1.1	1.2			1.1			
Egr1	1.3	1.3		1.3				1.2		
Maff	1.5	1.4	1.4	1.6						
Irf2	2.3	3.5	2.5	2.9				2.0		
Ets2	1.2			1.1			1.2			
Irf4		1.1		1.1			1.3			
Rela				1.2			1.1			
Jak2										
Runx1		1.3		1.3						
Hif1a				1.6			1.6			
Atf4				1.4				1.3		
E2f1							1.3			
Hsp90b1										
Ctcf										
Nek6										
Actb										
Ahr										
Ly96										

Perturb

	SCENIC	Pearson	MERLIN	PIDC	SCODE	LEAP	Scribe	Inferelator	kNN-DREMI	SILGGM
Number of predictable TFs	19	16	8	17	1	0	20	6	0	0
Atf3	1.4	1.3	1.2	1.6			1.5	1.2		
Rel	1.1	1.1	1.1	1.1			1.1			
Stat1	1.1	1.1		1.1			1.1	1.1		
Nfkb1	1.1	1.1	1.1	1.1				1.1		
Stat3	1.1	1.1		1.2			1.1			
Relb	1.3	1.1		1.2			1.3			
Stat2	1.1	1.1	1.1	1.1			1.1			
Egr2	1.2	1.1		1.3			1.3			
Cebpb	1.6	1.6	1.4	1.5			1.6	1.4		
Irf1	1.3	1.3	1.2	1.4			1.3			
Junb	1.4						1.5			
Egr1	1.7	1.3		1.7			2.2			
Maff	1.2	1.1	1.1							
Irf2							1.3			
Ets2	1.3	1.3		1.5			1.3			
Irf4	1.3	1.1		1.4			1.4			
Rela	1.1	1.1					1.2			
Jak2	2.2	2.1	1.8	2.6			1.9	2.9		
Runx1				1.1			1.2			
Hif1a	1.2						1.5			
Atf4										
E2f1							1.6			
Hsp90b1								13.2		
Ctcf							1.5			
Nek6					34.7					
Actb				3.5						
Ahr	1.3									
Ly96				11.6						

Number of predictable TFs

0.0	4.0	8.0	12.0	16.0	20.0

Fold enrichment

0.0	1.0	2.0	3.0	4.0	5.0

Figure S6. Predictable TFs for the Shalek dataset. Heatmaps show the enrichment of each transcription factor's target set from a perturbation-based (left) or ChIP-based (right) experimentally derived network in an inferred network. Columns are ordered based on overall ranking of methods. Individual white cells indicate transcription factors that were not considered as a predictable TF by a method. An entire row of white cells indicates the TF did not appear in one of the two experimentally derived networks.

	ChIP										Perturb									
	SCENIC	Pearson	MERLIN	PIDC	SCODE	LEAP	Scribe	Inferelator	kNN-DREMI	SILGGM	SCENIC	Pearson	MERLIN	PIDC	SCODE	LEAP	Scribe	Inferelator	kNN-DREMI	SILGGM
Number of predictable TFs	2	3	1	2	14	3	0	0	1	0	6	6	3	4	24	4	0	2	1	4
MSN2	2.8	1.9		2.5	4.4	2.0			2.7		2.3	2.1	2.2	3.1	2.1	2.1		2.1	3.5	
IFH1	2.9	7.7	4.0			5.0														
SFP1		4.7			3.3	4.8									1.6					
XBP1											4.5	3.9		7.0		4.8		4.3		
CIN5				1.9	3.8							6.2								
GIS1											4.1	3.9		5.1	3.6					
GCN4											1.7	1.7	1.8			1.6				
YAP1											2.0	2.4	2.1		2.2					
GCR1					15.9										3.6					
HSF1					4.2										3.8					
GCR2					6.5										5.7					
RAP1					2.3										4.9					
TYE7					9.9															8.1
STE12					2.5															
SOK2					2.7															
PDR1					3.0															
PHD1					2.1															
RPN4											2.6				2.5					
HFI1															6.0					4.8
FHL1					3.2															
SKO1					3.1															
SWI3															3.8					
RSF2															15.7					
MOT3															51.6					
AFT1															2.7					
TUP1															4.1					
MBF1																5.3				
SIN3															5.7					
REB1															5.8					
SNF6															3.6					
SNF2															3.9					
LEU3															5.0					
ABF1															3.0					
TEC1																				7.0
CUP2														7.3						
MGA2															3.1					
CST6															4.4					
SPT4																				1.6
HOG1															11.4					

Number of predictable TFs

0.0	4.0	8.0	12.0	16.0	20.0

Fold enrichment

0.0	1.0	2.0	3.0	4.0	5.0

Figure S7. Predictable TFs for the Gasch dataset. Heatmaps show the enrichment of each transcription factor's target set from a perturbation-based (left) or ChIP-based (right) experimentally derived network in an inferred network. Columns are ordered based on overall ranking of methods. Individual white cells indicate transcription factors that were not considered as a predictable TF by a method. An entire row of white cells indicates the TF did not appear in one of the two experimentally derived networks.

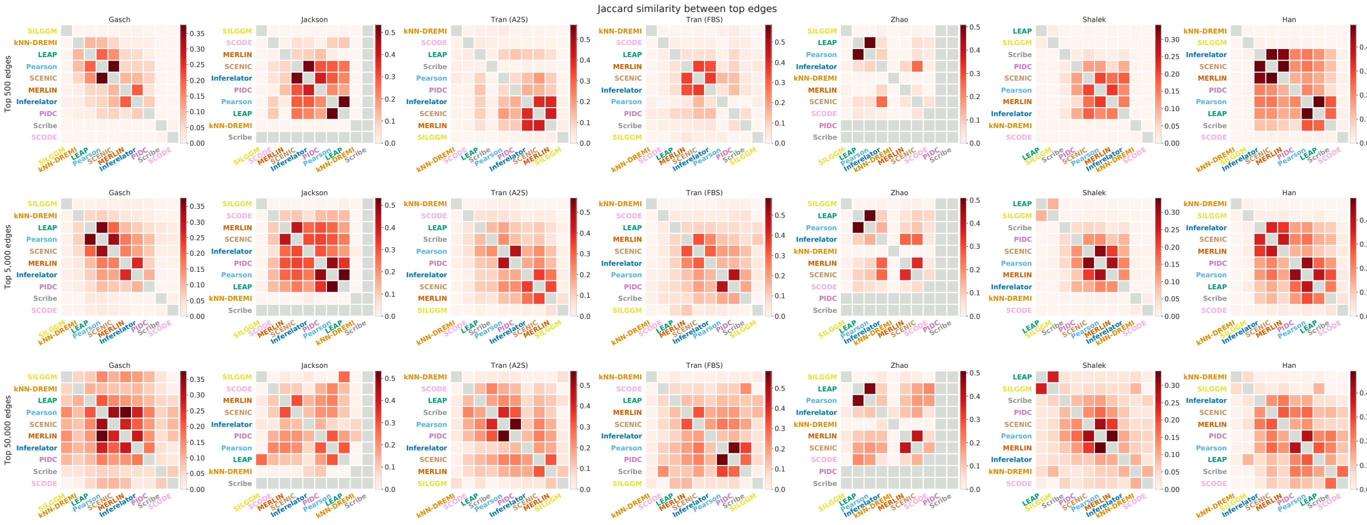
Jaccard similarity between top edges
Gasch
Jackson
Tran (A2S)
Tran (FBS)
Zhao
Shalek
Han
Top 500 edges
Top 5,000 edges
Top 50,000 edges

Figure S8. Jaccard similarity between top edge sets of inferred networks. Each heatmap showing inter-algorithm similarity between the networks inferred on each dataset (title), computed for the top 500, 5,000, and 50,000 edges. For each dataset, columns are ordered with respect to a hierarchical clustering of the similarity matrix based on the top 5,000 edges.

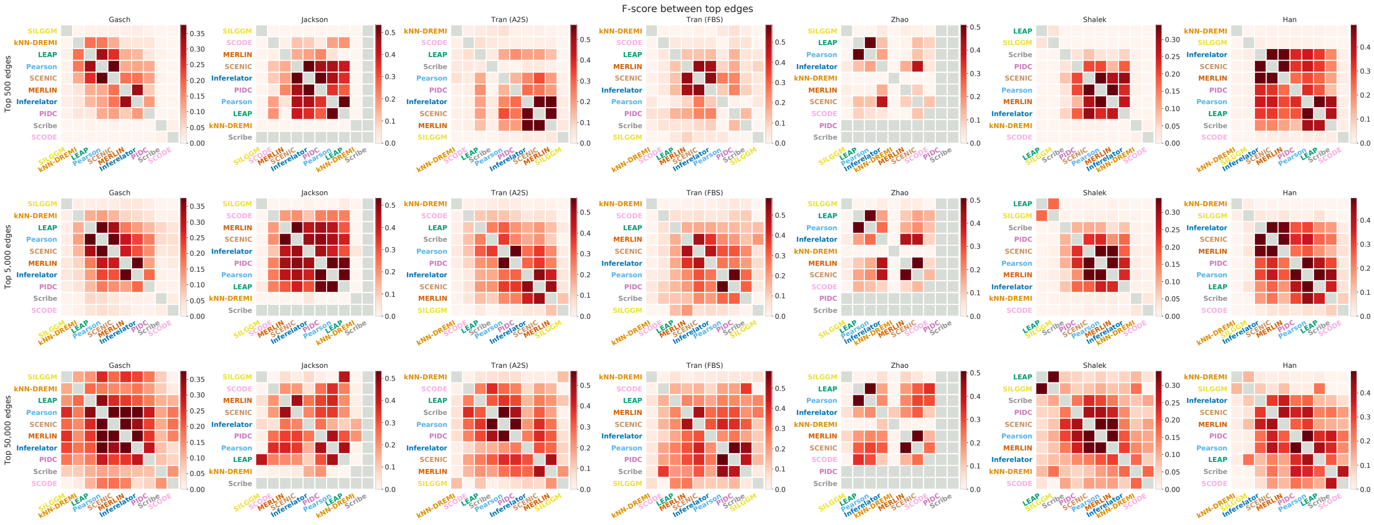

F-score between top edges
Gasch
Jackson
Tran (A2S)
Tran (FBS)
Zhao
Shalek
Han
Top 500 edges
Top 5,000 edges
Top 50,000 edges

Figure S9. F-score similarity between top edge sets of inferred networks. Heatmaps showing inter-algorithm similarity between the networks inferred on each dataset, computed for the top 500, 5,000, and 50,000 edges. For each dataset, columns are ordered with respect to a hierarchical clustering of the F-score similarity matrix based on the top 5,000 edges.

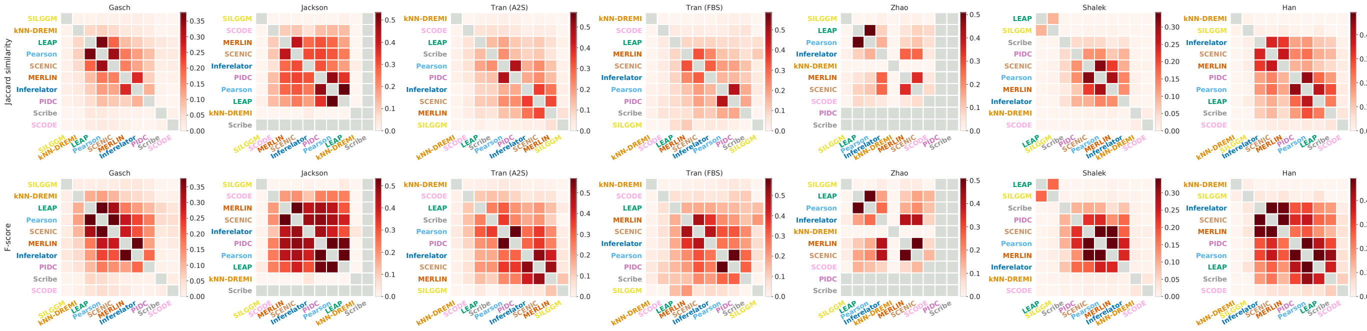

Gasch
Jackson
Tran (A2S)
Tran (FBS)
Zhao
Shalek
Han
Jaccard similarity
F-score
SILGGM
kNN-DREMI
LEAP
Pearson
SCENIC
MERLIN
Inferelator
PIDC
Scribe
SCODE

Figure S10. Comparison of Jaccard index and F-score as similarity metrics. Top, network similarity matrices using the Jaccard index for top 5k edges, with columns ordered by a hierarchical clustering of the respective matrix. Bottom, similarity matrices using the F-score. The patterns of network similarity are broadly similar when using either metric, while the F-score yields scores of higher magnitude.

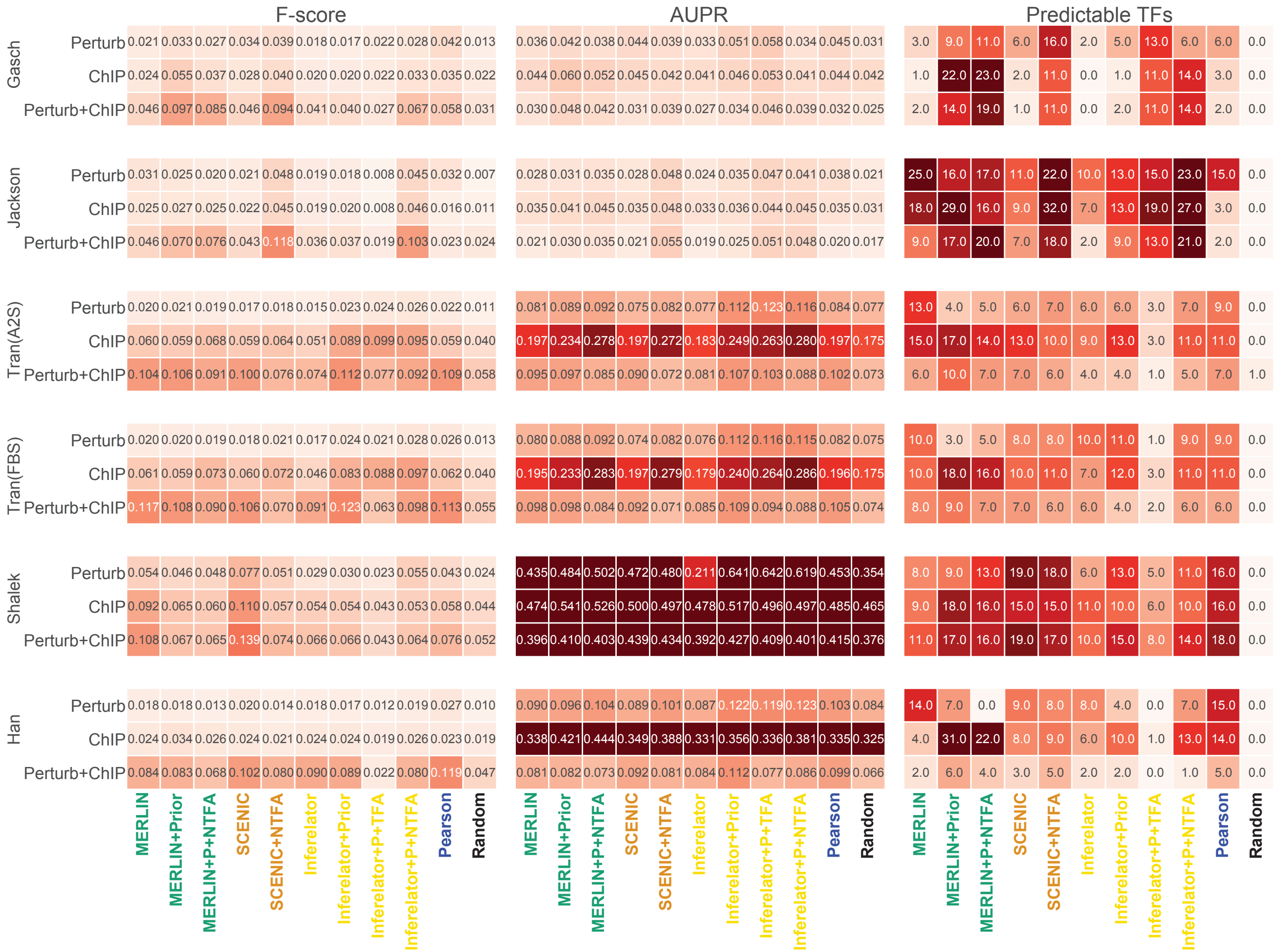
F-score
AUPR
Predictable TFs
Gasch
Jackson
Tran(A2S)
Tran(FBS)
Shalek
Han
Perturb
ChIP
Perturb+ChIP
0.021 0.033 0.027 0.034 0.039 0.018 0.017 0.022 0.028 0.042 0.013
0.024 0.055 0.037 0.028 0.040 0.020 0.020 0.022 0.033 0.035 0.022
0.046 0.097 0.085 0.046 0.094 0.041 0.040 0.027 0.067 0.058 0.031
0.031 0.025 0.020 0.021 0.048 0.019 0.018 0.008 0.045 0.032 0.007
0.025 0.027 0.025 0.022 0.045 0.019 0.020 0.008 0.046 0.016 0.011
0.046 0.070 0.076 0.043 0.118 0.036 0.037 0.019 0.103 0.023 0.024
0.020 0.021 0.019 0.017 0.018 0.015 0.023 0.024 0.026 0.022 0.011
0.060 0.059 0.068 0.059 0.064 0.051 0.089 0.099 0.095 0.059 0.040
0.104 0.106 0.091 0.100 0.076 0.074 0.112 0.077 0.092 0.109 0.058
0.020 0.020 0.019 0.018 0.021 0.017 0.024 0.021 0.028 0.026 0.013
0.061 0.059 0.073 0.060 0.072 0.046 0.083 0.088 0.097 0.062 0.040
0.117 0.108 0.090 0.106 0.070 0.091 0.123 0.063 0.098 0.113 0.055
0.054 0.046 0.048 0.077 0.051 0.029 0.030 0.023 0.055 0.043 0.024
0.092 0.065 0.060 0.110 0.057 0.054 0.054 0.043 0.053 0.058 0.044
0.108 0.067 0.065 0.139 0.074 0.066 0.066 0.043 0.064 0.076 0.052
0.018 0.018 0.013 0.020 0.014 0.018 0.017 0.012 0.019 0.027 0.010
0.024 0.034 0.026 0.024 0.021 0.024 0.024 0.019 0.026 0.023 0.019
0.084 0.083 0.068 0.102 0.080 0.090 0.089 0.022 0.080 0.119 0.047
0.036 0.042 0.038 0.044 0.039 0.033 0.051 0.058 0.034 0.045 0.031
0.044 0.060 0.052 0.045 0.042 0.041 0.046 0.053 0.041 0.044 0.042
0.030 0.048 0.042 0.031 0.039 0.027 0.034 0.046 0.039 0.032 0.025
0.028 0.031 0.035 0.028 0.048 0.024 0.035 0.047 0.041 0.038 0.021
0.035 0.041 0.045 0.035 0.048 0.033 0.036 0.044 0.045 0.035 0.031
0.021 0.030 0.035 0.021 0.055 0.019 0.025 0.051 0.048 0.020 0.017
0.081 0.089 0.092 0.075 0.082 0.077 0.112 0.123 0.116 0.084 0.077
0.197 0.234 0.278 0.197 0.272 0.183 0.249 0.263 0.280 0.197 0.175
0.095 0.097 0.085 0.090 0.072 0.081 0.107 0.103 0.088 0.102 0.073
0.080 0.088 0.092 0.074 0.082 0.076 0.112 0.116 0.115 0.082 0.075
0.195 0.233 0.283 0.197 0.279 0.179 0.240 0.264 0.286 0.196 0.175
0.098 0.098 0.084 0.092 0.071 0.085 0.109 0.094 0.088 0.105 0.074
0.435 0.484 0.502 0.472 0.480 0.211 0.641 0.642 0.619 0.453 0.354
0.474 0.541 0.526 0.500 0.497 0.478 0.517 0.496 0.497 0.485 0.465
0.396 0.410 0.403 0.439 0.434 0.392 0.427 0.409 0.401 0.415 0.376
0.090 0.096 0.104 0.089 0.101 0.087 0.122 0.119 0.123 0.103 0.084
0.338 0.421 0.444 0.349 0.388 0.331 0.356 0.336 0.381 0.335 0.325
0.081 0.082 0.073 0.092 0.081 0.084 0.112 0.077 0.086 0.099 0.066
3.0 9.0 11.0 6.0 16.0 2.0 5.0 13.0 6.0 6.0 0.0
1.0 22.0 23.0 2.0 11.0 0.0 1.0 11.0 14.0 3.0 0.0
2.0 14.0 19.0 1.0 11.0 0.0 2.0 11.0 14.0 2.0 0.0
25.0 16.0 17.0 11.0 22.0 10.0 13.0 15.0 23.0 15.0 0.0
18.0 29.0 16.0 9.0 32.0 7.0 13.0 19.0 27.0 3.0 0.0
9.0 17.0 20.0 7.0 18.0 2.0 9.0 13.0 21.0 2.0 0.0
13.0 4.0 5.0 6.0 7.0 6.0 6.0 3.0 7.0 9.0 0.0
15.0 17.0 14.0 13.0 10.0 9.0 13.0 3.0 11.0 11.0 0.0
6.0 10.0 7.0 7.0 6.0 4.0 4.0 1.0 5.0 7.0 1.0
10.0 3.0 5.0 8.0 8.0 10.0 11.0 1.0 9.0 9.0 0.0
10.0 18.0 16.0 10.0 11.0 7.0 12.0 3.0 11.0 11.0 0.0
8.0 9.0 7.0 7.0 6.0 6.0 4.0 2.0 6.0 6.0 0.0
8.0 9.0 13.0 19.0 18.0 6.0 13.0 5.0 11.0 16.0 0.0
9.0 18.0 16.0 15.0 15.0 11.0 10.0 6.0 10.0 16.0 0.0
11.0 17.0 16.0 19.0 17.0 10.0 15.0 8.0 14.0 18.0 0.0
14.0 7.0 0.0 9.0 8.0 8.0 4.0 0.0 7.0 15.0 0.0
4.0 31.0 22.0 8.0 9.0 6.0 10.0 1.0 13.0 14.0 0.0
2.0 6.0 4.0 3.0 5.0 2.0 2.0 0.0 1.0 5.0 0.0
MERLIN
MERLIN+Prior
MERLIN+P+NTFA
SCENIC
SCENIC+NTFA
Inferelator
Inferelator+Prior
Inferelator+P+TFA
Inferelator+P+NTFA
Pearson
Random

Figure S11. Network inference utilizing gene expression alone compared to addition of priors and Transcription factor Activity (TFA). The terms "+P" after an algorithm's name denotes adding prior while addition of "+TFA" or "+NTFA" corresponds to TFA estimated using Inferelator's inbuilt TFA estimation or TFA estimated from Network Components Analysis (NTFA), respectively. The performance of MERLIN, SCENIC, and Inferelator is reported along with their enhanced versions incorporating prior, TFA or both, on 6 datasets. On the left are the values for F-score. The middle column shows values for AUPR. On the right are the number of predictable TFs. For calculating the F-score and predictable TFs we considered only the top 5,000 edges while for AUPR we used all edges. We also report these metrics for Pearson and the Random network.
